# Contrast enhanced ultrasound imaging of the optic nerve sheath diameter – what are we really measuring?

**DOI:** 10.1186/2036-7902-4-S1-A2

**Published:** 2012-12-18

**Authors:** Andrej Bergauer, G Prosen, V Flis, T Šeruga, M Brvar, N Kobilica

**Affiliations:** 1 Department for Vascular Surgery, University Clinical Center Maribor, Ljubljanska 5, Maribor, Slovenia; 2 Center for Emergency Medicine, Ulica talcev 9, Maribor, Slovenia; 3 Department for Radiology, University Clinical Center Maribor, Ljubljanska 5, Maribor, Slovenia

## Background

Several studies on pediatric and adult population proposed measurment of optic nerve sheath diameter (ONSD) as a noninvasive marker of increased intracranial pressure (ICP). Only few studies were made correlating MRI measurment of ONSD with ultrasound measurment of ONSD. Overall lower standard values of the ONSD for ultrasound measurment compared to MRI measurment were found. That might be atribbuted to variable interpretation of ultrasound anatomy – what are we really measuring?

## Objective

We performed a proof of concept study to evaluate the accuracy of measurments of the ONSD for contrast enhanced ultrasound (CEUS) and magnetic resonance imaging (MRI). Second generation contrast agent (Sonovue™, Bracco SpA) was used to enhance the ultrasound recognition of relevant anatomy and conduct transbulbar ONSD measurments.

## Patients and methods

Nine healthy volunteers were examined with CEUS with transbulbar approach and MRI. CEUS and MRI examinations were recorded on the PACS system. Measurments of the ONSD were performed on the collected images using DICOM viewing software (OsiriX™, Pixmeo SARL). Statistical analysis was performed and included the calculation of the agreement of measurment between both methods. Statistical software was used (IBM SPSS Statistics ver. 20™, IBM Corp).

## Results

Good correlation of measurment values was found between CEUS and MRI (ICC 0.98, 95% CI, 0.74 – 0.99), MRI being regarded as a gold standard.

## Conclusion

Using CEUS significantly aids the identification and recognition of the relevant structures sorrounding the optic nerve. Measuring a small structure as ONSD with ultrasound is a demanding task. By using CEUS the exact measuring points can be quickly and easilly identified, making a measurment more exact using transbulbar sonography on living subjects. The measurment can be quickly performed, can be repeated, the introduced contrast agent is nontoxic
